# Broadening the identification of superior cognition in older age

**DOI:** 10.1055/s-0043-1764451

**Published:** 2023-03-22

**Authors:** Emily Rogalski

**Affiliations:** 1 Northwestern University, Feinberg School of Medicine, Mesulam Center for Cognitive Neurology and Alzheimer's Disease, Chicago, Illinois, United States.; 2 Northwestern University, Feinberg School of Medicine, Department of Psychiatry and Behavioral Sciences, Chicago, Illinois, United States.

The renewed enthusiasm in identifying factors contributing to youthful or unusually successful cognitive aging holds promise for understanding factors important for optimizing healthspan and avoiding Alzheimer's disease and related disorders. Likewise, studies focused on better-than-expected memory performance in older age provide important paradigms for informing mechanisms of reserve, resilience, resistance, and compensation (e.g.). 1 2 3 4 5 6 7 8 Carmona and colleagues 9 extend this work by drawing from the population-based Pietà Study in Brazil to explore whether “high-performance older adults (HPOA)” are present and to identify potential sociodemographic, clinical, and lifestyle features that set them apart from typical agers (called “standard performance older adults (SPOA).”

Of the 132 individuals included, 18 (13.6%) met HPOA criteria, suggesting it is a relatively uncommon occurrence. Consistent with previous SuperAging studies, more women than men met criteria; however, longevity of women relative to men and research participation bias may be contributors. The HPOA and SPOA individuals were compared on several factors and found to differ only on two, age and depression symptoms. HPOA individuals were younger than SPOA individuals and endorsed fewer depression symptoms on the 15-item Geriatric Depression Scale (GDS). The age-related result reinforces the concept of superior cognition as an index of resilience and resistance becomes more meaningful with age. 10

A particularly novel aspect of this report was the identification of HPOA individuals with low education. Education and more recently quality of education have been identified as key risk factors for Alzheimer's disease. Here, the proof-of-concept presence of HPOA individuals with low education holds promise for understanding mechanisms for resilience in individuals who have been historically underrepresented in research. Likewise, the existence of high performers with low education provides opportunity for reflection and scrutiny of previously established associations, allowing for nuanced revisions and interpretations as new knowledge emerges.

There is tremendous opportunity for improving our understanding of risk versus protective factors in aging, especially in diverse cohorts. Do the pathways for maintaining superior performance in older age differ by education level, race/ethnicity, or sex? Which of these factors are modifiable? Carmona and colleagues 9 have embraced this approach and provided an initial indication that superior memory performance in older age is possible even with low education. This exciting prospect deserves further investigation to uncover significant biologic, psychosocial, genetic, and other contributors, and may benefit from prospectively designed collaborative science designs. One example of such an effort is the SuperAging Research Initiative, a multisite consortium established in 2021, which is focused on identifying factors promoting youthful cognitive aging by increasing minority representation and expanding deep phenotyping of this unique phenotype. 11 Investment in global initiatives may help disentangle generalizable versus cohort-specific pathways relevant for promoting extended healthspan.
